# Supplementary material for: Estimating the early death toll of COVID-19 in the United States
Source: medRxiv. 2020 Apr 29:2020.04.15.20066431. Originally published 2020 Apr 18. Preprint. [Version 2] doi: 10.1101/2020.04.15.20066431 (PMC7217085; doi:10.1101/2020.04.15.20066431)
Supplement: 1 [file NIHPP2020.04.15.20066431-supplement-1.pdf]

medRxiv preprint doi: https://doi.org/10.1101/2020.04.15.20066431; this version posted April 29, 2020. The copyright holder for this preprint (which was not certified by peer review) is the author/funder, who has granted medRxiv a license to display the preprint in perpetuity.
It is made available under a CC-BY 4.0 International license .

Supplement to: Estimating the early death toll of COVID-19 in the United States

Daniel M. Weinberger, PhD Ted Cohen, MD DPH, Forrest W. Crawford PhD, Farzad Mostashari MD, Don Olson MPH, Virginia E Pitzer ScD, Nicholas G Reich PhD, Marcus Russi BS, Lone Simonsen PhD, Anne Watkins BS, Cecile Viboud PhD

SUPPLEMENTAL FIGURES 2
SUPPLEMENTAL TABLES 5
SUPPLEMENTAL METHODS 8

medRxiv preprint doi: https://doi.org/10.1101/2020.04.15.20066431; this version posted April 29, 2020. The copyright holder for this preprint
(which was not certified by peer review) is the author/funder, who has granted medRxiv a license to display the preprint in perpetuity.
It is made available under a CC-BY 4.0 International license .

SUPPLEMENTAL FIGURES

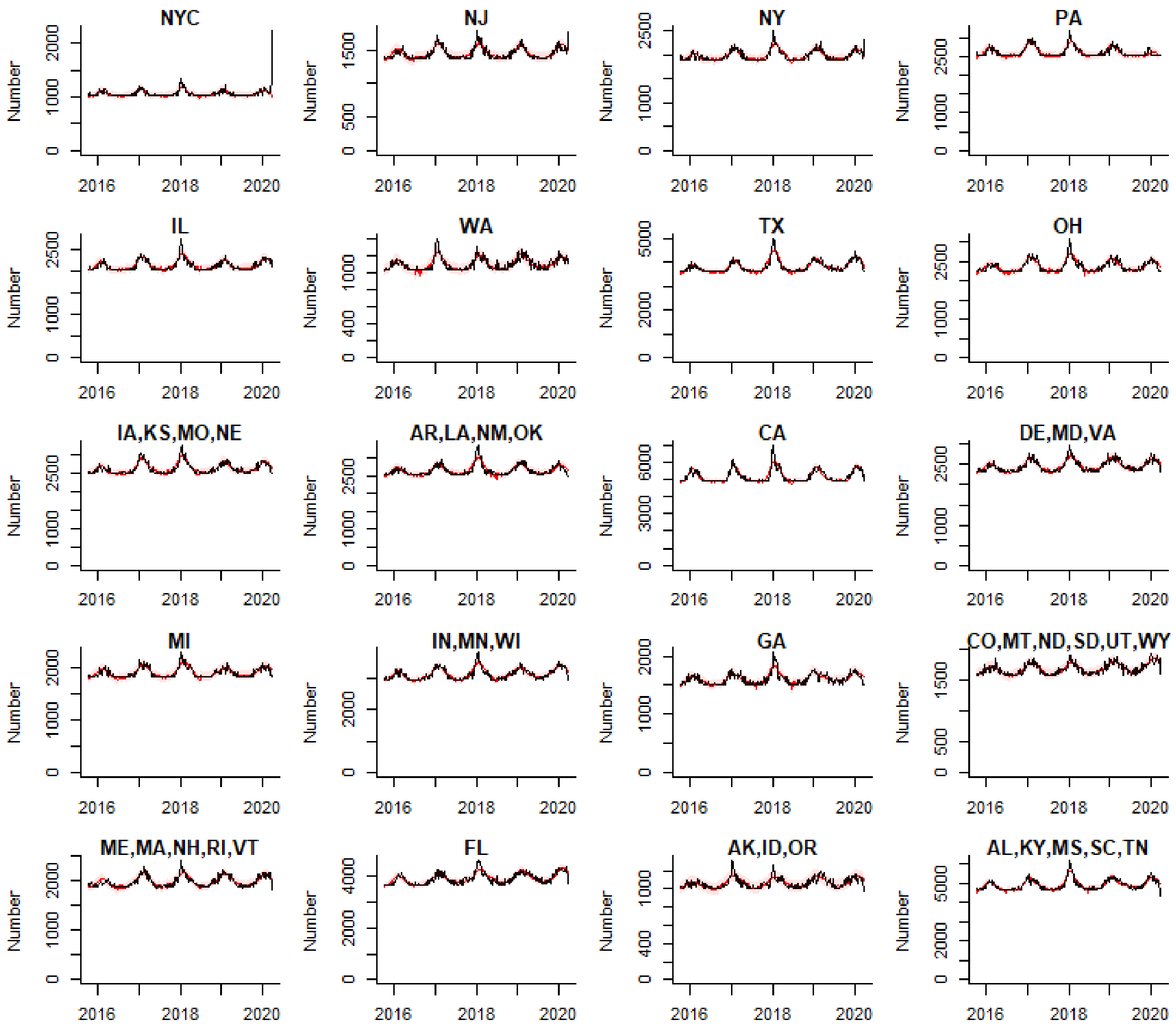

Figure S1: Excess all-cause deaths. The black line shows the observed number of *all* deaths per week, regardless of cause. The red line and shaded area represent the 95% Prediction Interval. The latest data is for the week ending 2020-03-28. Note that these are adjusted for percent completeness of the data using the NCHS' estimate of data completeness. There are clear jumps in all-cause mortality in NY and NJ, other states are stable or decreasing, likely due to reporting delays.

medRxiv preprint doi: https://doi.org/10.1101/2020.04.15.20066431; this version posted April 29, 2020. The copyright holder for this preprint
(which was not certified by peer review) is the author/funder, who has granted medRxiv a license to display the preprint in perpetuity.
It is made available under a CC-BY 4.0 International license .

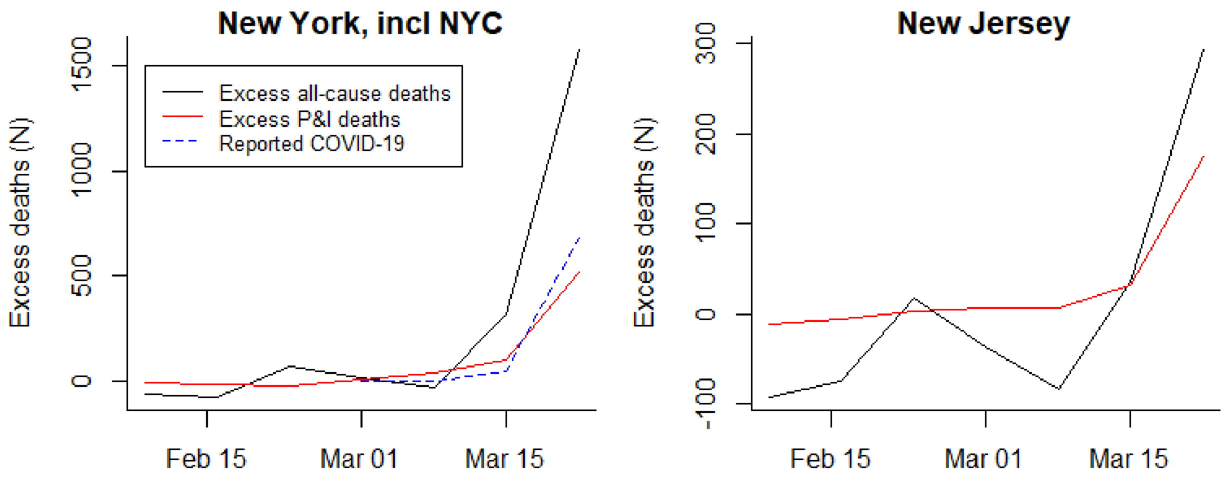

Figure S2: Excess all-cause deaths (black) vs Excess deaths due to pneumonia and influenza (red) and reported COVID-19 deaths from the COVID Tracking Project (blue dashed line) For New York (including New York City) and New Jersey.

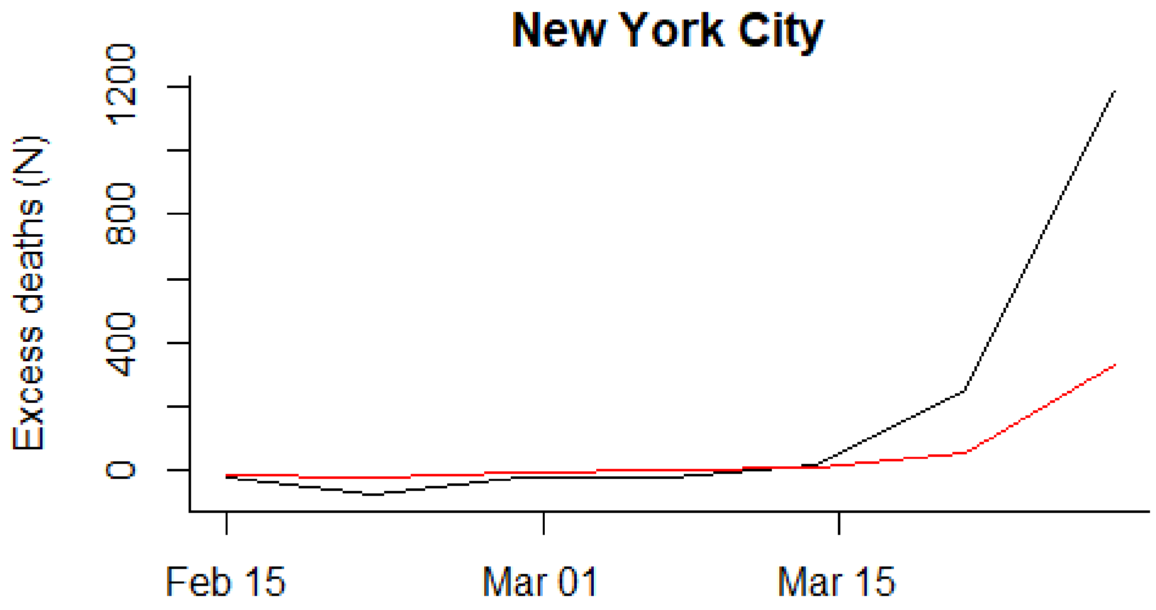

Figure S3. Excess P&I deaths per week (red) vs all-cause excess deaths (black) in New York City only.

medRxiv preprint doi: https://doi.org/10.1101/2020.04.15.20066431; this version posted April 29, 2020. The copyright holder for this preprint (which was not certified by peer review) is the author/funder, who has granted medRxiv a license to display the preprint in perpetuity.
It is made available under a CC-BY 4.0 International license .

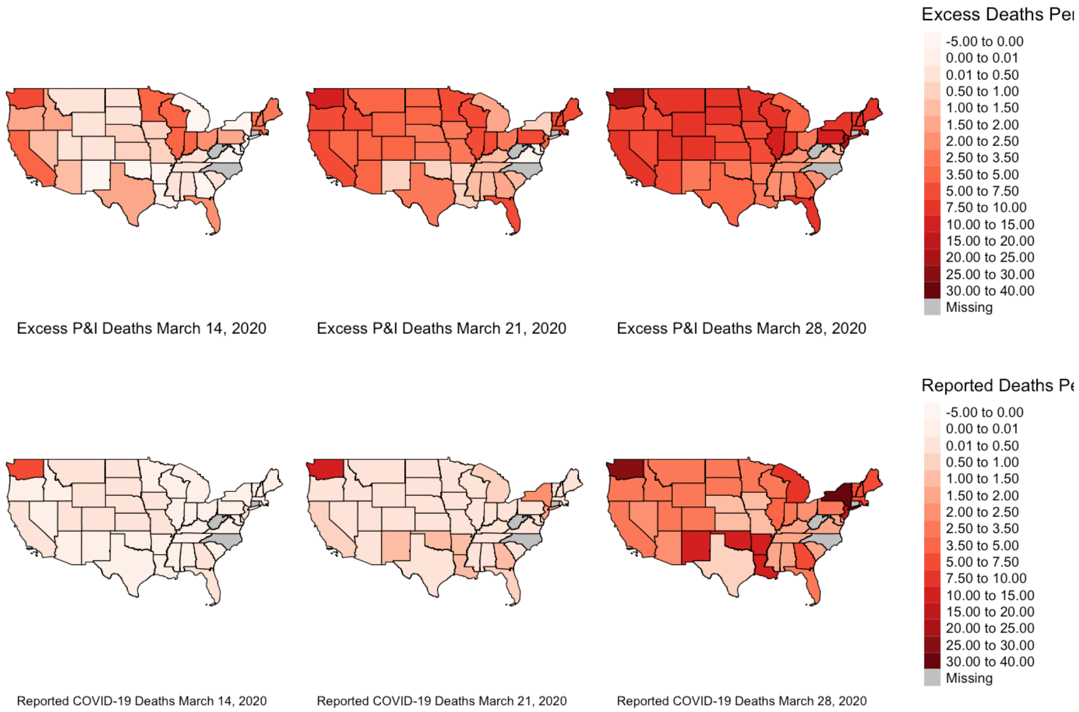

Figure S4. Map of Excess deaths by state and COVID-19 deaths reported by The COVID Tracking Project

medRxiv preprint doi: https://doi.org/10.1101/2020.04.15.20066431; this version posted April 29, 2020. The copyright holder for this preprint (which was not certified by peer review) is the author/funder, who has granted medRxiv a license to display the preprint in perpetuity.
It is made available under a CC-BY 4.0 International license .

SUPPLEMENTAL TABLES

Table S1: Comparison of data sources: Observed and Excess deaths due to pneumonia & influenza, and COVID-19, from February 9, 2020 through Mar 28, 2020

Week ending	Total P&I deaths	Excess P&I deaths	NCHS Reported COVID-19 Deaths, with pneumonia code	NCHS Reported COVID-19 Deaths	All reported COVID-19 deaths (covidtracking.com)
2020-02-15	3971	-71(-202, 60)	0	0	0
2020-02-22	3867	-132(-262, -2)	0	0	0
2020-02-29	3998	67(-62, 196)	3	5	1
2020-03-07	4092	270(143, 397)	11	19	22
2020-03-14	4046	455(332, 578)	22	44	36
2020-03-21	4317	887(767, 1007)	200	435	229
2020-03-28	4877	1622(1505, 1739)	928	2034	1670

medRxiv preprint doi: https://doi.org/10.1101/2020.04.15.20066431; this version posted April 29, 2020. The copyright holder for this preprint
(which was not certified by peer review) is the author/funder, who has granted medRxiv a license to display the preprint in perpetuity.
It is made available under a CC-BY 4.0 International license .

Table S2: Proportion of COVID-19 deaths with a pneumonia code, by state, through most recent date. Note these values will be greater than those in other tables, which are 2 weeks behind

State	NCHS Reported COVID-19 Deaths	NCHS Reported COVID-19 Deaths, with pneumonia code	Proportion with pneumonia
Total US	6930	3161	0.46
Alabama	17	6	0.35
Alaska	1	1	1
Arizona	26	13	0.5
Arkansas	10	2	0.2
California	187	102	0.55
Colorado	107	60	0.56
Delaware	2	2	1
District of Columbia	5	5	1
Florida	157	93	0.59
Georgia	74	42	0.57
Hawaii	1	0	0
Idaho	13	4	0.31
Illinois	77	47	0.61
Indiana	43	19	0.44
Iowa	12	2	0.17
Kansas	9	3	0.33
Kentucky	6	3	0.5
Louisiana	154	64	0.42
Maine	10	5	0.5
Maryland	75	42	0.56
Massachusetts	172	85	0.49
Michigan	309	142	0.46
Minnesota	17	6	0.35
Mississippi	34	17	0.5
Missouri	22	9	0.41
Montana	3	1	0.33
Nebraska	3	2	0.67
Nevada	27	21	0.78
New Hampshire	15	8	0.53
New Jersey	593	303	0.51
New Mexico	1	0	0
New York	1130	650	0.58
New York City	2862	1065	0.37
North Dakota	1	0	0
Ohio	8	4	0.5
Oklahoma	17	5	0.29
Oregon	21	13	0.62
Pennsylvania	220	98	0.45
Rhode Island	1	1	1
South Carolina	36	13	0.36
South Dakota	4	1	0.25
Tennessee	25	11	0.44
Texas	47	18	0.38
Utah	8	3	0.38
Vermont	17	6	0.35
Virginia	39	15	0.38
Washington	278	142	0.51
Wisconsin	34	7	0.21
Puerto Rico	18	13	0.72

medRxiv preprint doi: https://doi.org/10.1101/2020.04.15.20066431; this version posted April 29, 2020. The copyright holder for this preprint (which was not certified by peer review) is the author/funder, who has granted medRxiv a license to display the preprint in perpetuity.
It is made available under a CC-BY 4.0 International license .

Table S3. Comparison of baselines that are or are not adjusted for influenza. Observed and Excess deaths due to pneumonia & influenza, and COVID-19, from February 9, 2020 through March 28, 2020.

State	Excess P&I deaths (unadjusted)	Excess P&I deaths (adjusted)	Reported COVID-19 deaths
NY	620(526, 714)	620(526, 714)	728
CA	393(280, 506)	399(287, 512)	101
NJ	206(154, 258)	206(154, 258)	140
WA	187(139, 235)	187(139, 235)	207
IL	185(117, 253)	185(117, 253)	47
FL	180(93, 267)	180(93, 267)	54
TX	159(71, 247)	176(88, 264)	27
PA	156(96, 216)	182(122, 242)	34
IN,MN,WI	146(62, 230)	150(66, 234)	49
ME,MA,NH,RI,VT	110(40, 180)	114(44, 183)	59
IA,KS,MO,NE	110(35, 185)	124(48, 199)	20
CO,MT,ND,SD,UT,WY	101(41, 161)	105(46, 165)	36
AR,LA,NM,OK	74(3, 145)	95(24, 166)	158
DE,MD,VA	65(-4, 134)	67(-2, 136)	25
AZ,HI,NV	61(-1, 123)	62(0, 125)	25
GA	47(-2, 96)	68(20, 117)	69
AK,ID,OR	45(5, 85)	51(11, 92)	19
AL,KY,MS,SC,TN	38(-65, 141)	47(-57, 150)	43
MI	37(-27, 101)	37(-27, 101)	92
OH	34(-23, 91)	44(-14, 101)	25

medRxiv preprint doi: https://doi.org/10.1101/2020.04.15.20066431; this version posted April 29, 2020. The copyright holder for this preprint (which was not certified by peer review) is the author/funder, who has granted medRxiv a license to display the preprint in perpetuity.
It is made available under a CC-BY 4.0 International license .

Table S4. Comparison of estimates when the estimates for excess cases are adjusted based on the estimated completeness of the database. Excess deaths due to pneumonia & influenza from February 9, 2020 through Mar 28, 2020 with or without adjustment for delayed reporting

state	Excess P&I (unadjusted)	Excess P&I (adjusted for reporting delay)
NY	514(420, 608)	620(526, 714)
CA	370(257, 482)	399(287, 512)
NJ	206(154, 258)	206(154, 258)
WA	156(108, 204)	187(139, 235)
IL	165(97, 233)	185(117, 253)
PA	75(15, 135)	182(122, 242)
FL	179(92, 266)	180(93, 267)
TX	88(0, 177)	176(88, 264)
IN,MN,WI	101(17, 185)	150(66, 234)
IA,KS,MO,NE	62(-13, 137)	124(48, 199)
ME,MA,NH,RI,VT	61(-9, 130)	114(44, 183)
CO,MT,ND,SD,UT,WY	53(-7, 113)	105(46, 165)
AR,LA,NM,OK	67(-5, 138)	95(24, 166)
GA	40(-9, 88)	68(20, 117)
DE,MD,VA	39(-30, 108)	67(-2, 136)
AZ,HI,NV	46(-16, 109)	62(0, 125)
AK,ID,OR	17(-23, 58)	51(11, 92)
AL,KY,MS,SC,TN	42(-61, 145)	47(-57, 150)
OH	13(-44, 70)	44(-14, 101)
MI	46(-18, 110)	37(-27, 101)

SUPPLEMENTAL METHODS

Statistical model

We developed a regression model for P&I deaths in epidemiological year *i* (July-June) and week *t* as a function of seasonal parameters and the percent positive influenza tests in the prior week. Models were fit separately for each location with data from January 5, 2015 to February 8, 2020; fitted values were projected for the period until March 28, 2020. Let $PI\_Deaths_{i,t}$ be the number of P&I deaths and let $Flu\_Pct\_Pos_{i,t-1}$ be the percent positive influenza tests. We modeled

$$PI\_Deaths_{i,t} \sim Poisson(\boldsymbol{\lambda}_{i,t})$$

where

$$\log(\boldsymbol{\lambda}_{i,t}/Total\_death_{i,t}) = \boldsymbol{\beta}_0 + \boldsymbol{\beta}_1 * \sin(\Theta_t) + \boldsymbol{\beta}_2 * \cos(\Theta_t) + \boldsymbol{\beta}_3 * \sin(\Theta_t/2) + \boldsymbol{\beta}_4 * \cos(\Theta_t/2) + + \boldsymbol{\beta}_6 * \log(Flu\_Pct\_Pos_{i,t-1}) + \gamma_i + \alpha_i * \log(Flu\_Pct\_Pos_{i,t-1})$$

and

$$\Theta_t = 2 * \boldsymbol{\pi} * t/52.1775$$

To compute prediction intervals, we used the following procedure. Once the regression coefficients were estimated, we extracted the estimated asymptotic covariance matrix for the parameters and constructed a multivariate normal distribution approximating the sampling distribution, centered at the estimated parameter values. We drew 100 samples from this parameter distribution, computed the resulting mean value $\boldsymbol{\lambda}_{i,t}$, and then drew 100 samples from

medRxiv preprint doi: https://doi.org/10.1101/2020.04.15.20066431; this version posted April 29, 2020. The copyright holder for this preprint
(which was not certified by peer review) is the author/funder, who has granted medRxiv a license to display the preprint in perpetuity.
It is made available under a CC-BY 4.0 International license .

the Poisson distribution with this mean. This resulted in 10,000 samples from an empirical predictive distribution of $PI\_Deaths_{i,t}$. Empirical 95% prediction intervals were computed by taking the 2.5th and 97.5th percentiles of this resulting distribution. In further sensitivity analyses, we evaluated a model in which Flu_Pct_Pos was excluded altogether.
